# Breakdown of One-to-One Correspondence in Energy and Volume in a High-Pressure Heat-Treated Zr-Based Metallic Glass During Annealing

**DOI:** 10.1038/s41598-020-64442-1

**Published:** 2020-05-04

**Authors:** Rui Yamada, Yuki Shibazaki, Yasuto Abe, Wookha Ryu, Junji Saida

**Affiliations:** 1 Frontier Research Institute for Interdisciplinary Sciences (FRIS), Tohoku University, Sendai, 980-8578 Japan; 2 International Centre for Young Scientists (ICYS), National Institute for Materials Science (NIMS), Tsukuba, 305-0044 Japan

**Keywords:** Engineering, Materials science

## Abstract

The glassy state of a high-pressure heat-treated Zr_50_Cu_40_Al_10_ metallic glass was investigated from energetic and volumetric perspectives. The specific heat (*C*_*p*_) data of initial heating and subsequent cooling from the supercooled liquid region indicates that the sample behaviour cannot be explained simply by the derivative of the well-known enthalpy (*H*)–temperature (*T*) curve. Unlike the *C*_*p*_ data, the thermal-expansion coefficient (*α*) value increased monotonically during the first heating step, which suggests the collapse of a one-to-one correspondence between the energy and volume during the process. The *α* data of the cooling process follow almost the same path as those of the as-cast process, whereas the corresponding *C*_*p*_ curves do not. This result implies that the volume appears to be more sensitive to obeying external heat compared with energy, which highlights the different time scale for achieving an equilibrium state in energy and volume. The second heating data of the *C*_*p*_ and *α* exhibit an inverse relationship against the corresponding first heating set, which confirms the breakdown of a one-to-one correspondence during annealing. The newly constructed energy–density diagram shows that the treated sample is rejuvenated volumetrically but is relaxed energetically during aging, which has never been observed experimentally previously.

## Introduction

High-pressure behaviour has been studied intensively in the metallic glass field. Many interesting phenomena, such as polyamorphism^1,2^, liquid-to-liquid transition^3^, and pressure-induced devitrification^4,5^, were found by applying a pressure of the gigapascal order with/without heat treatment to several metallic glass alloy systems. Our previous study revealed that an abnormal glassy state, which is thought to be difficult to prepare only by annealing of the as-cast sample under ambient pressure, appears to form in high-pressure heat-treated Zr_50_Cu_40_Al_10_ metallic glass^6^. Also, it was confirmed that the sample possessed excellent mechanical properties (*i.e*., a high strength with some ductility)^6^. Although several anomalous signs were detected (*e.g*., ultra-dense packing, which was denser than the partially crystallized state), the decisive evidence of creating such an unusual glassy state remains to be clarified.

**Figure 1:**
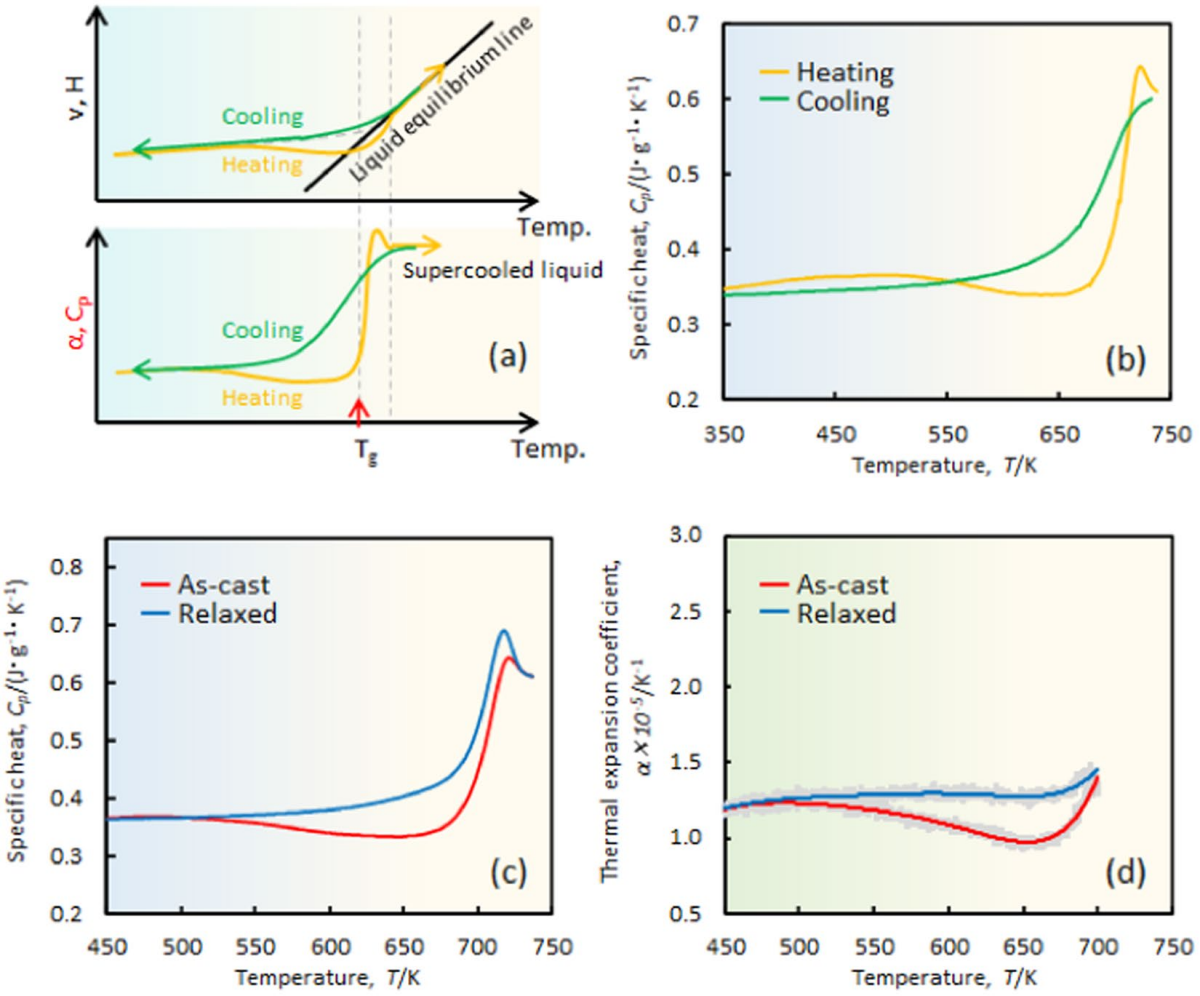
(**a**) Schematic illustration of [volume (*v*), enthalpy (*E*)]–temperature (*T*) and its derivative {*i.e*., [specific heat (*C*_*p*_), thermal expansion coefficient (*α*)]–temperature(*T*)} diagrams for general glasses. (**b**) *C*_*p*_ curves of as-cast Zr_50_Cu_40_Al_10_ metallic glass obtained during heating and cooling. (**c**) *C*_*p*_ and (**d**) corresponding *α* curves of as-cast and statically annealed (relaxed) samples.

In general, the glassy state is characterized by the glass volume/energy^7,8^. The upper illustration in Fig. 1(a) shows a well-known theoretical (volume (*v*), enthalpy (*H*))–temperature (*T*) diagram of the glassy materials. The vertical axis is normally described as either the volume (*v*) or enthalpy (*H*)^7,8^, which means that an almost one-to-one correspondence exists between energy and volume (*i.e*., a higher energy state had a higher volume, and vice versa). The relationship is thought to be universal for typical glassy materials^7,8^ and the previous reports suggested that it is also applicable for the metallic glass alloy systems^9–11^.

In early glass research, Angell claimed the importance of measuring the derivative thermodynamic properties when investigating the glassy state^12,13^. Thermodynamically, derivative/integrate relationships exist between the enthalpy (*H*)/specific heat (*C*_*p*_) and volume (*v*)/thermal-expansion coefficient (*α*), as described by:

1 $${C}_{p}=\frac{dH}{dT},\,\alpha =\frac{1}{v}\frac{dv}{dT}$$

where *T* is the temperature. A measurement of these thermodynamic parameters enables a clarification of the actual glassy nature of the glass. As mentioned previously, because the energy and volume should show a one-to-one correspondence, it is sufficient to measure only one of these parameters. In general, the specific heat is easier to measure (*e.g*., less shape restriction, a few milligrams is sufficient to measure), therefore, *C*_*p*_ is used extensively (*e.g*., especially in relaxation/rejuvenation studies^8,14–16^) to discuss the glassy nature thus far.

The creation of eccentric glass with high-pressure heat treatment, whose glassy state is thought to be difficult to define through a conventional manner (*i.e*., does not obey the one-to-one correspondence between energy and volume), was proposed in a molecular dynamic (MD) simulation^17–20^. For example, Ding *et al*. found an inverse volume–energy relationship for the pressure-mediated metallic glass^17^. Miyazaki *et al*. reported the creation of a high-density, well-ordered, but high-energy metallic glass that was designed by pressurized quenching^18,19^. Although these studies were not conducted under the same simulation conditions and the initial material state of applying high-pressure heat treatment were different (*e.g*., the former was for the melt, whereas the latter was for the once-created glassy solid), the obtained results were similar. Accordingly, a breakdown of one-to-one correspondence between energy and volume was observed by the simulation studies.

This background indicates that it is important to investigate the relationship between the energy and volume of the high-pressure heat-treated sample. Therefore, the measurement of *C*_*p*_ and *α* are necessary to clarify the glassy state. Empirically, a specimen size of a few millimetres, especially, a height of at most around 5 mm is feasible to measure *α* precisely. Because the collecting of a bulk sample with such a height under the high pressure with heat treatment is difficult, only a few reports exist with a thorough discussion on the glassy nature of the high-pressure heat-treated state^21^.

In our previous study, we successfully collected a bulk sample with a height of ~8 mm of Zr_50_Cu_40_Al_10_ metallic glass with a heat-treated state by using belt type large volume press apparatus combining with quenching process^6^. Then, we evaluated several material properties (*e.g*., density, electric resistivity, and mechanical properties) of the sample quantatively^6^. By extending the evaluation field to the *C*_*p*_ (*i.e*., energy term) and *α* (*i.e*., volume term) measurements, it was possible to reveal the high-pressure heat-treated state experimentally. The outcomes could provide beneficial information for the high-pressure heat-treatment effect on random atomic configuration systems and a perspective to develop a new series of glassy states. Also, it may give a clue to elucidate the relationship between the glassy state and heterogeneity of the glass, whose topic is still under discussion^22,23^. Hence, it is important to identify the created state.

In this study, we investigated the energy–volume relationship of a high-pressure heat-treated (HPHTed) sample through specific-heat and thermal-expansion coefficient measurements during annealing and density data to reveal the glassy nature of the created state and demonstrate the possibility to develop new types of glassy states that are inaccessible at ambient conditions.

## Results

### Observation of breakdown of one-to-one correspondence in energy and volume during annealing

Figure 1(b) shows specific heat curves of the as-cast sample that were obtained during heating and cooling. The shapes of the curves are similar to the one, which is predicted from a differentiation of the enthalpy (*H)*–temperature (*T)* curves [described in Fig. 1(a) below]. This result proved that the metallic glasses follow the *H*–*T* diagram during heating and cooling.

Figure 1(c,d) show the specific-heat and thermal-expansion-coefficient curves of the as-cast and relaxed states. The shapes of those curves are relatively close and the positions of the corresponding states match. For example, when the glass shows a high specific-heat value, the thermal-expansion coefficient exhibits a high value, and vice versa. These results show strong evidence of a one-to-one correspondence between energy and volume in normal metallic glasses. Although the curves of α should trace a similar path with *C*_*p*_, no α data exist above the glass transition temperature (*i.e*., *T*_*g*_~700 K) (Fig. 1(d)), due to the difficulties in obtaining data in the vicinity of *T*_*g*_ because the expansion was detected by using a probe in the dilatometer and the sample softened significantly near *T*_*g*_. The same problem has been identified in a previous paper^24^.

**Figure 2:**
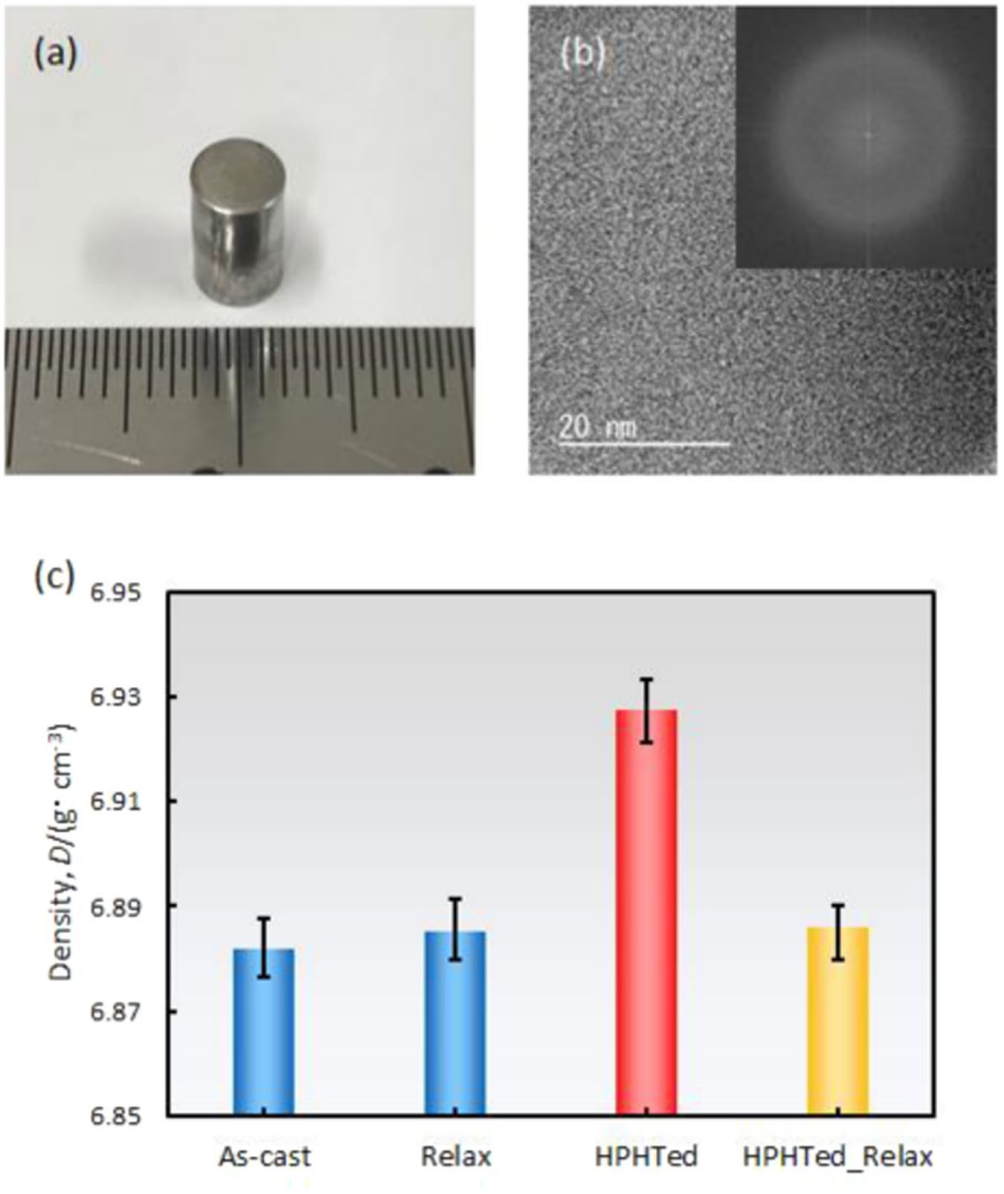
(**a**) Outer appearance of collected bulk sample after high-pressure heat treatment. (**b**) High-resolution transmission electron microscope image and its FFT. No signals that correspond to a crystalline state were detected. (**c**) Measured density data at room temperature of each glassy state.

Figure 2(a) shows the outer appearance of a collected bulk sample with a high-pressure heat treatment. The sample maintains a bulk rod shape that enables us to measure the specific-heat and thermal-expansion coefficient. Figure 2(b) shows a high-resolution transmission electron microscope image of the collected sample. No contrasts that correspond to the crystalline phase are visible. Furthermore, no crystalline spots in the fast Fourier transformation image (Fig. 2(c)) indicate that the sample maintains a fully amorphous structure. The microstructure was also observed with X-ray diffractometry and the result also shows the fully amorphous characteristic (see our previous report^6^).

**Figure 3:**
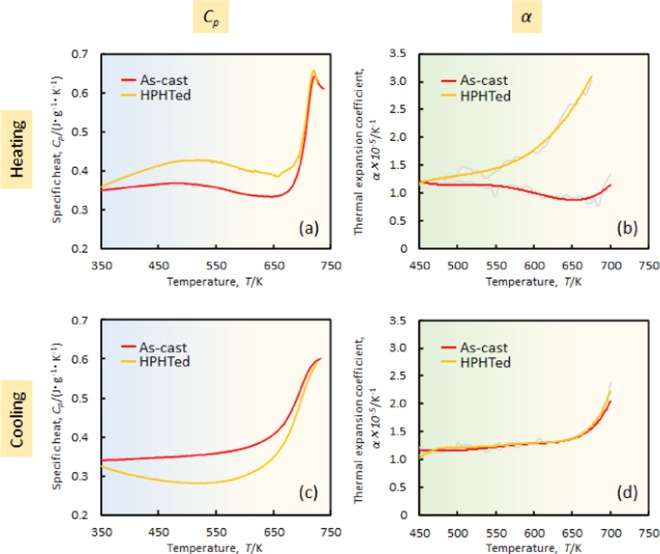
*C*_*p*_ [(**a**,**c**)] and *α* [(**b**,**d**)] curves of as-cast and HPHTed sample. (**a**) and (**b**) First and (**c**) and (**d**) second cooling process.

Figure 3(a) shows the specific heat curves of the as-cast and HPHTed sample. The specific heat value of the HPHTed sample is larger than the as-cast sample over the entire temperature range. The large difference is especially visible below 700 K. The relatively large endothermic broad signal prior to the glass transition (temperature range between 350 K and 650 K) is visible in the HPHTed curve. This result suggests that a relaxed state is created in the high-pressure heat-treated sample.

Figure 3(b) exhibits the thermal-expansion coefficient of each state. For the as-cast sample, a large decrease of *α* is visible, which starts from ~550 K and ends at 670 K, and occurs because of the structural relaxation during heating. In the temperature range, atoms receive energy from the environment to rearrange the atomic configuration, which leads to an annihilation of the free volume. Closer to the supercooled liquid region (above 670 K), the α starts to increase, which corresponds to an introduction of the free volume. Interestingly, unlike the as-cast sample, a pronounced increase of *α* is observed in the HPHTed sample. This distinct increase is incomparable with that of the static annealed state (*i.e*., relaxed state (see Fig. 1(d)). For instance, the *α* value of the relaxed sample at 650 K is ~1.3 × 10^−5^ K^−1^, whereas it is approximately two times larger (*i.e*. ~2.6 × 10^−5^ K^−1^) for the HPHTed sample. Such a large *α* is thought to have originated from the strong repulsive force between atoms during heating. The high-pressure forces shorten the atomic distance, and the denser state is frozen by quenching from 850 K. During heating, the thermal vibration is activated so that the atoms can move with a large repulsive force, which results in a significant expansion of the atomic distances. Unlike the *C*_*p*_ data (Fig. 3(a)), which show an increase and a decrease, the monotonous increase of *α* with temperature is observed. This result appears to suggest that a one-to-one correspondence between energy and volume fails during heating.

Figure 3(c) presents cooling curves of the *C*_*p*_ of the as-cast and HPHTed samples from the supercooled liquid region. As indicated in Fig. 1(b), the data for the as-cast sample are similar to the theoretically predicted curve. For the HPHTed sample, the curve decreased sharply to 550 K and then increased. This behaviour cannot be explained by considering the derivative of the conventional *H*–*T* curve (lower illustration in Fig. 1(a)), which suggests the creation of an anomalous glassy state in the sample. Figure 3(d) shows *α* data of the cooling of each state. The two curves nearly overlap, which indicates that the thermal-expansion coefficients on cooling are almost identical. Because the cooling curves of *C*_*p*_ are different between the HPHTed and as-cast sample, the thermal-expansion measurement results suggests the presence of an inconsistent behaviour of energy and volume during annealing. Large differences between *C*_*p*_ and *α*, which have been, to the best of our knowledge, observed for the first time, are thought to originate from a high-pressure heat-treatment effect on the glassy state. The details of such large differences are discussed later.

**Figure 4:**
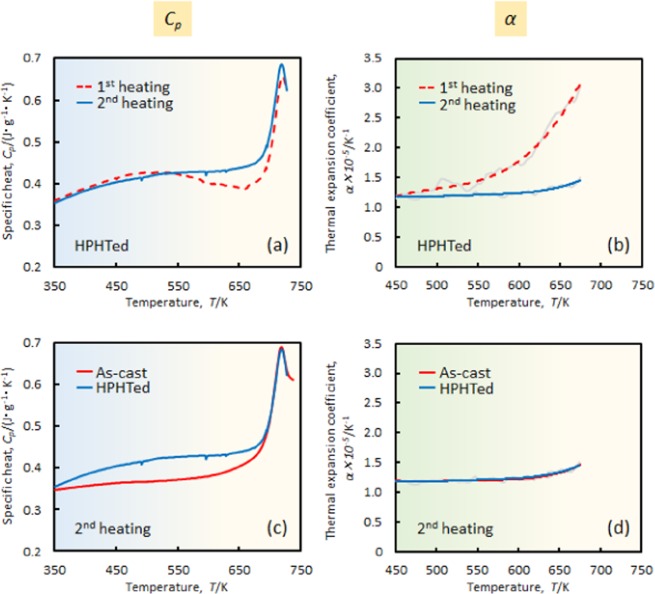
(**a**) *C*_*p*_ and (**b**) *α* curves of first and second heating steps of HPHTed sample. (**c**) *C*_*p*_ and (**d**) *α* curves of second heating step of as-cast and HPHTed sample.

Figure 4(a) shows the *C*_*p*_ data of the HPHTed sample of the second and first heating curves. Although a slightly lower value is observed until 530 K, the second heating curve is mostly placed above the first heating curve. The enthalpy relaxation, whose signal appears around the peak of the *C*_*p*_^25^ (720 K), is more pronounced for the second heating curve, which suggests the achievement of a more relaxed state after annealing in the supercooled liquid region. Figure 4(c) presents the second heating curves of the *C*_*p*_ data of the as-cast and HPHTed samples. The curves do not trace each other. The *α* data of the second heating step of the HPHTed sample is shown in Fig. 4(b) (first heating data are inserted as a reference). The curve shows a relatively constant value of 1.2–1.5 × 10^−5^ K^−1^. Interestingly, the position order of the corresponding curves in Fig. 4(a,b) is reversed. Accordingly, the *C*_*p*_ value of the second heating step is mostly higher than the first heating step, whereas it exhibits an opposite relationship for the *α*. As shown in Fig. 1(c,d), the order of the corresponding curves for the statically annealed glass are consistent. These results support the perspective of a breakdown of a one-to-one correspondence between energy and volume during annealing. Figure 3(d) indicates that the volume states of the as-cast and HPHTed samples after annealing in the supercooled liquid region may unify. To confirm the prediction, the *α* data of the second heating step were measured, and they are described in Fig. 4(d). Unlike the energy data (see Fig. 4(c)), the curves trace almost the same path, which is indicative of the uniform volume state in each sample.

### Construction of the energy-density diagram

**Figure 5:**
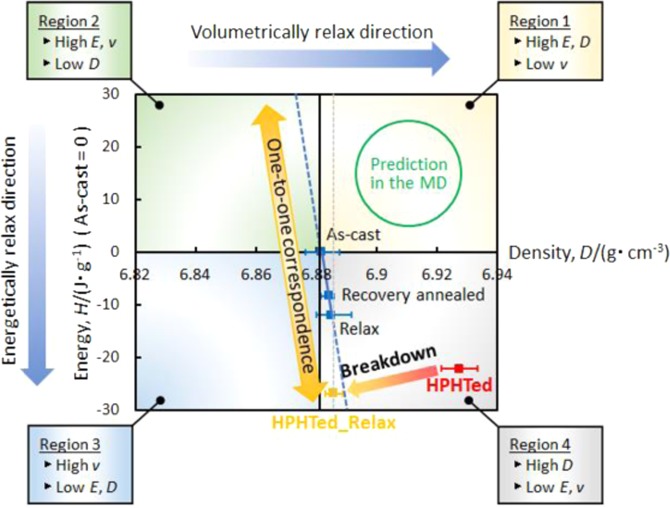
Energy–density diagram of each state (as-cast, relaxed, recovery annealed, HPHTed and HPHTed_Relax states). Vertical axis derived from energy difference against as-cast state. Assuming a linear relationship, a one-to-one correspondence in energy and volume is described as the blue line.

It is important to clarify whether the high-energy, high-density metallic glass, whose state is predicted in the MD simulation result can be created via high-pressure heat treatment. The relationship between the energy and density of the HPHTed Zr_50_Cu_40_Al_10_ metallic glass were investigated. Figure 5 shows the energy-density diagram of the Zr_50_Cu_40_Al_10_ metallic glass in each glassy state. Here, the standard zero point in the energy (*i.e*., vertical axis) is derived from the as-cast data. The energy state of each sample was calculated from an integration of *ΔC*_*p*_ data that were surrounded by the as-cast curve. For example, the relaxation enthalpy of the HPHTed sample (*ΔH*_*HPHTed*_) was calculated from (*i.e*., the surrounding area of the two *C*_*p*_ curves in Fig. 3(a)):

2 $$\varDelta {H}_{HPHTed}={\int }_{RT}^{{T}_{a}}({C}_{p,HPHTed}-{C}_{p,as-cast})dT$$

where RT and *T*_*a*_ are room, annealing temperatures (*e.g*., for the HPHTed sample, the temperature is 730 K) and *C*_*p,HPHTed*_ and *C*_*p,as-cast*_ are the specific heats of the HPHTed and as-cast samples. The calculated value was *ΔH*_*HPHTed*_ = 22.2 J/g. Since the *C*_*p*_ curve of the HPHTed sample places above the one for the as-cast sample [see Fig. 3(a)], the HPHTed sample exhibits 22.2 J/g lower energy state (*i.e*. −22.2 J/g) than the as-cast state. The crystallization enthalpy of the as-cast sample is *ΔH*_*cry*_ = 45.9 J/g^6^, hence, the energy level of the HPHTed state is *ΔH*_*HPHTed*_*/ΔH*_*cry*_ × 100 = 48% of that of the crystallized state. It was confirmed in our density measurement that the HPHTed sample shows a high density of 6.928 g/cm^3^, which is ~0.6% higher than that (6.882 g/cm^3^) of the as-cast state^6^ (see Fig. 2(c)). The details of the enthalpy and density values of each state are summarized in Table 1. As mentioned previously, a one-to-one correspondence (here, for sake of simplicity, the linear relation is assumed and it is described by the solid blue line in Fig. 5) is visible in the as-cast, relaxed and recovery annealed (see Methods section) sample (*i.e*., a lower energy yields a denser glass). The high energy with high-density glass, which is expected in the MD simulation results^18,19^, is located in Region 1. The obtained experimental data in this study are plotted in Fig. 2(c). The data point exists in Region 4. In contrast with the MD simulation prediction, the HPHTed sample exhibits a significantly relaxed state from an energetic and volumetric perspective. The results indicate that the high-energy with high-density metallic glass is not created through the high-pressure heat treatment.

**Table 1:** Measured density and enthalpy data of each glassy state.

	Density, D (g·cm^−3^)	Enthalpy, *ΔH* (J·g^−1^) (As-cast = 0)
As-cast	6.882	0
Relax	6.885	−11.97
Recovery annealed	6.884	−8.31
HPHTed	6.928	−22.2
HPHTed_Relax	6.886	−26.9

Although the created glass is confirmed to be low-energy with an ultra-dense state, the collapse of a one-to-one correspondence during annealing is suggested from our *C*_*p*_ and *α* data. The relaxed state of the HPHTed sample (HPHTed_Relax) was prepared by annealing at ambient pressure, and we measured its density (see Fig. 2(c)). The obtained density was 6.886 g/cm^3^, which is lower than the initial state (6.928 g/cm^3^) and is almost the same as that (6.885 g/cm^3^) of the relaxed sample (*i.e*., annealed as-cast sample at ambient pressure). This result proves our prediction that the volume state of the HPHTed sample that was annealed in the supercooled liquid region is recovered.

The cooling rate of 20 K/min from the supercooled liquid region tends to be sufficiently slow to relax the sample energetically and volumetrically. The energy/density states of the annealed sample tend to be lower/higher than the initial state (See Table 1). In contrast with the statically relaxed sample, the HPHTed_Relax state shows a less-dense state than the initial state, even though the cooling rate was slow. The sample enthalpy was calculated by substituting *C*_*p, HPHTed*_ into *C*_*p, HPHTed_Relax*_ (which was obtained from the second heating curve in Fig. 4(a)) in Eq. (2). The value was 26.9 J/g, which indicates that an energetically more relaxed state than the as-received HPHTed (*ΔH*_*HPHTed*_ = 22.2 J/g) was presented. The obtained density and enthalpy data were plotted in Fig. 5. The extrapolated line of the as-received (*i.e*., HPHTed) and relaxed (*i.e*., HPHTed_Relax) data are nearly perpendicular to the one-to-one correspondence line (solid blue line). Therefore, the breakdown of one-to-one correspondence between energy and volume was confirmed to have occurred during annealing of HPHTed sample. It is worth mentioning that we observed a unique phenomenon, that is, an energetic relaxation and, at the same time, volumetric rejuvenation through normal annealing.

The observed breakdown phenomenon is totally different from the previously reported one^26^. Ge *et al*. have reported that the higher density with higher energy state was successfully attained after high-pressure treatment of the Ce_65_Al_20_Cu_10_Co_5_ based metallic glass^26^. In our case, the higher density with lower energy state was firstly observed in the as-received HPHTed sample (the data point of the HPHTed sample locates in Region 4 in Fig. 5). However, during annealing process, the reverse relationship between energy and volume was surely observed. To the best of our knowledge, it is the first report on a “dynamic” breakdown (*i.e*. here, “dynamic” means “during annealing”), which is clearly distinguished by the previously observed one^26^ (so to call “static” breakdown).

## Discussion

Dmowski *et al*. investigated the reversibility of the structure of the high-pressure quenched (HPQ) sample through synchrotron X-ray data^3^. They annealed the HPQ sample at ambient pressure and 5 K below the glass transition temperature (*T*_*g*_ –5 K) for 5 min and compared the annealed HPQ sample with the as-cast sample that was annealed at the same thermal conditions. The difference in the pair distribution functions *G*(r) is small and it can be concluded that the original structure is recovered substantially by annealing. In our study, as discussed in Fig. 2(c), 3(d) and 4(d), the glassy state is recovered volumetrically by annealing in the supercooled liquid region. Although the annealing temperature and time differ (*i.e*., they are below the *T*_*g*_ for 5 min, whereas in our case they are 20~30K above the *T*_*g*_ for 2 min), the same tendency of the recovery of the state is observed. The *G*(r) data tend to reflect the atomic distance information and correspond to the volume data of our result. In contrast, as described in Fig. 4(c), the energy term does not recover completely through annealing. The origin of such different behaviours in volume and energy are thought to be derived from “topological” and “chemical” effects on the atomic ordering. The volume and energy terms appear to be related to the former and latter, respectively. In the volume term, a spatial atomic configuration is related, where an introduction/annihilation of the free volume is concerned, whereas, in the energy term, the chemical bonding pair is thought to be associated strongly, such as a medium-/short-range order.

The second heating curves of the as-cast and high-pressure heat-treated sample in *C*_*p*_ overlapped above 700 K (Fig. 4(c)). It is suggested that either/both the annealing temperature and/or time in the supercooled liquid is sufficiently energetic to achieve the equilibrium state, and therefore, their energy state over *T*_*g*_ is almost identical. If so, the energy state below *T*_*g*_ should also be the same, because their activation energies are normally lower than those around *T*_*g*_. Indeed, even though the initial energy states differ, once the glass is annealed in the supercooled liquid region and held for a sufficient time, they achieve almost the same energy state over the temperature range (see Supplementary Fig. S1). In the same analogy, it is believed that the *C*_*p*_ curves of the as-cast and HPHTed samples should overlap after annealing in the supercooled liquid region. However, as mentioned previously, the second heating curves differ at a lower *T*_*g*_ (*i.e*., a broad endothermic signal was observed for the HPHTed sample compared with the as-cast sample). To make the broad signal disappear, a longer annealing time of 5 min at 730 K was employed. However, the signal did not vanish through the annealing (see Supplementary Fig. S2). These results may suggest that the relaxation mechanism with a strong chemical bonding, which is thought to be difficult to make disappear only for annealing at ambient pressure for several minutes, existed in the system.

One can predict that the highly ordered relaxation mechanism is introduced in the system through high-pressure heat treatment. Indeed, a previous MD simulation result suggested that a well-ordered atomic configuration is promoted by the treatment^18,19^. An appearance of the endothermic signal in *C*_*p*_ during heating, which is observed from 350 K to 650 K in Fig. 3(a), indicates that the introduced relaxation mechanism can be related to the chemical short-range order (CSRO). The CSRO is observed frequently in low-temperature annealing in metallic glasses with a low *T*_*g*_^27,28^. As shown in Supplementary Fig. S3(a), the broad signal, which has been recognized as the excess endothermic reaction because of the decomposition of the CSRO that was formed^29^, was observed at ~400 K (denoted by arrows) in the room-temperature relaxed Pd_42.5_Cu_30_Ni_7.5_P_20_ sample. For the Zr_50_Cu_40_Al_10_ systems, which show a relatively high *T*_*g*_ ~700 K, the endothermic signal is visible around 650 K (denoted by arrows in Supplementary Fig. S3(b)) through annealing at 585 K (~0.83*T*_*g*_) for 7 h. As Evenson *et al*. observed through positron annihilation lifetime spectroscopy (PALS) and high-intensity synchrotron X-ray scattering measurements^28^, the changes in CSRO may not be accompanied by a reduction in overall free volume, which can be interpreted broadly as the breakdown of a one-to-one correspondence in volume and energy. Slipenyuk *et al*. claimed that the enthalpy change during relaxation is always (low- and high-temperature relaxation) accompanied by a free-volume reduction and concluded that a linear dependence exists between the enthalpy change and free volume change^30^ (*i.e*., suggestion of a one-to-one correspondence in energy and volume). Supplementary Fig. S3(b) shows that the corresponding signal that was observed in the endothermic peak in *C*_*p*_ at ~650 K does not appear in the *α* curve, which shows that the one-to-one correspondence is breakdown in a CSRO. If so, one would assume that the HPHTed state corresponds to the CSRO-formed structure, because the phenomenon is similar, accordingly, an endothermic signal in *C*_*p*_ appears and the possibility of breakdown occurs of one-to-one correspondence in energy and volume during annealing. However, both CSRO signals that were observed in Supplementary Fig. S3(a,b) were erased after annealing in the supercooled liquid region for only 2 min. Although the signal decreases slightly with an increase in annealing time, the observed broad endothermic signal in the HPHTed sample still exists through annealing for more than 2 min. Both endothermic signals are related to the CSRO in Supplementary Fig. S3(a,b) appear above the annealing temperature (*T*_*a*_) (*i.e*., for the Pd_42.5_Cu_30_Ni_7.5_P_20_ sample, *T*_*a*_ is room temperature (~300 K) and the endothermic signal appears at 400 K (100 K above *T*_*a*_). For the Zr_50_Cu_40_Al_10_ sample, *T*_*a*_ is 585 K and the signal appears at 650 K (65 K above *T*_*a*_)). The peak of the endothermic signal for the HPHTed sample is ~510 K, which is below the treated temperature (*T*_*a*_ = 850 K) at 5.5 GPa. These behaviours differ from what was observed in the CSRO. Therefore, it is too early to interpret the introduced relaxation mechanism in the HPHTed state as the CSRO at this point. Although the extensively ordered relaxation mechanism appears to be present in the treated sample, further investigation (*e.g*. dynamic mechanical analysis *etc*.) is required to identify details of the structural origin of the state. It will give a useful information on the relationship between the glassy state and structural heterogeneity^22^.

As described in Supplementary Fig. S2, annealing for 5 min in the supercooled liquid region is insufficient to vanish the endothermic signal in *C*_*p*_, whereas for the *α* measurement, it is sufficient to follow the liquid equilibrium state. These results imply that the volume is more sensitive to obey external heat compared with energy. Therefore, the different time scales to achieve the state in energy and volume was predicted.

Figure 5 shows almost the same volume/density (DHPHTed_Relax/DRelax = 1.001) but different energy states (HHPHTed_Relax/HRelax = 3.237) were created through annealing of the HPHTed sample for the first time. The creation of such an abnormal state will overturn the fundamental glass concept that has been accepted widely thus far. The well-known (volume (*v*), enthalpy (*H*))–temperature (*T*) diagram is invalid in terms of explaining the HPHTed state during annealing. The diagram is thought to be common for  typical glass formers (though an exception can be observed for CSRO, which is described in Supplementary Fig. S3(b,c)), because there is an assumption that the relationship between volume and energy follows a one-to-one correspondence in an ambient-pressure environment. However, because our results reveal the breakdown in relationship during annealing in the HPHTed sample, the diagram needs be separated into two different descriptions (*i.e*., volume (*v*)–temperature (*T*) and enthalpy (*H*)–temperature (*T*)) in a specific case. Also, it provides a new interpretation to explain several phenomena that remain under discussions. A clarification of the origin of the fracture behaviour is conceivable. Thus far, it remains uncertain as to whether the fracture is governed mainly by the free volume (topological) and/or atomic bonding (chemical) contributions. By using the Relax and HPHTed_Relax samples, accordingly, the volume state is almost the same, but the energy state is different, it can be predicted which terms primarily govern the phenomenon. Also, our newly created state will help clarify the crystallization and corrosion mechanism, which are thought frequently to be governed by the amount of free volume^31^,^32^ thus far. Therefore, our findings will illuminate the creation of a new series of glassy states and provide beneficial information to disclose several physical phenomena.

## Methods

Alloy ingots with a nominal composition of Zr_50_Cu_40_Al_10_ were prepared by arc melting a mixture of each pure element in an argon atmosphere. The 4-mm-diameter metallic glassy rod samples were prepared by tilt casting and they were cut to an 8-mm height. The amorphous structure was examined by X-ray diffractometry with CuK_α_ radiation (X-ray diffractometry, Rigaku MultiFlex) and transmission electron microscopy (TEM, JEOL JEM-2000F) observation. The HPHTed state was obtained by using a belt-type large-volume press apparatus installed at National Institute for Materials Science (NIMS), Japan. First, the rod sample was compressed quasi-isotropically with an applied pressure of 5.5 GPa at room temperature. Then, the sample was heated with a heating rate of 20 K/min at 850 K, which is near but remains below the crystallization temperature (*T*_*x, 5.5 GPa*_ ~870 K) for 2 min followed by rapid cooling (~100 K/s). After cooling, the bulk sample was collected to measure the specific heat (*C*_*p*_), thermal-expansion coefficient (*α*) and density. The as-cast, relaxed (annealing the as-cast state at ~1.05*T*_*g*_ = 740 K for 2 min followed by 20 K/min cooling) and recovery annealed (annealing the relaxed state at 740 K for 2 min followed by ~280 K/min cooling) samples were prepared as references. The *C*_*p*_ were measured by DSC (Perkin Elmer Diamond DSC). Each sample was heated to an annealing temperature (*T*_*a*_), which is in the supercooled liquid region (first heating step), and held for 2 min followed by 20 K/min cooling (first cooling step). The sample was heated again to *T*_*a*_ (second heating step) to measure the *C*_*p*_ and *α*. *T*_*a*_ was set at 740 K for the as-cast, relaxed, recovery annealed samples, whereas it was 730 K for the HPHTed sample. In any process, the heating rate was set at 20 K/min. The thermal-expansion coefficients of each state were measured with a dilatometer (NETZSCH, DIL402C) and the thermal-mechanical analysis (TMA, TA Instruments, Q400) apparatus. The former was used for the as-cast and relaxed samples in Fig. 1(d) whereas the latter was used for the HPHTed and as-cast samples (Figs. 3 and 4). The heating and cooling rates of each machine were set at 5 K/min. For the TMA measurement, annealing temperatures of 733 and 723 K were set for the as-cast and HPHTed sample, respectively. The heating and cooling were considered in the *C*_*p*_ and *α* measurements, which has never been discussed previously, to discuss the created glassy nature that was annealed in the supercooled liquid region. The relaxed state (annealed at 730 K for 2 min followed by 20 K/min cooling) of the HPHTed sample (*i.e*., HPHTed_Relax) was prepared to measure the density. The density of each state was measured with a gas pycnometer (SHIMADZU micromeritics AccuPycII 1340). The data were obtained by averaging 30 measurement points. The density was measured by using Archimedes’ method as standard data.

## Supplementary information

Supplementary information.
