# Supplementary material for: Breakdown of One-to-One Correspondence in Energy and Volume in a High-Pressure Heat-Treated Zr-Based Metallic Glass During Annealing
Source: Sci Rep. 2020 May 4;10:7438. doi: 10.1038/s41598-020-64442-1 (PMC7198604; doi:10.1038/s41598-020-64442-1)
Supplement: Supplementary file 1 — Supplementary information. [file 41598_2020_64442_MOESM1_ESM.docx]

Supplementary Figure S1


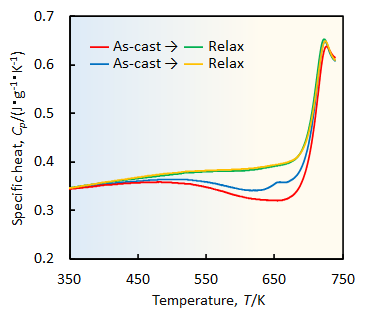


Supplementary Figure S2


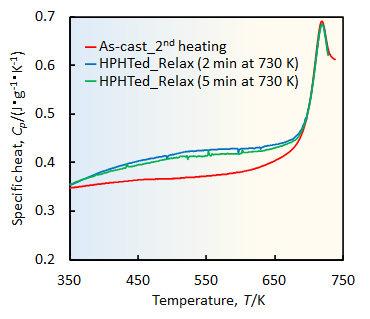


Supplementary Figure S3


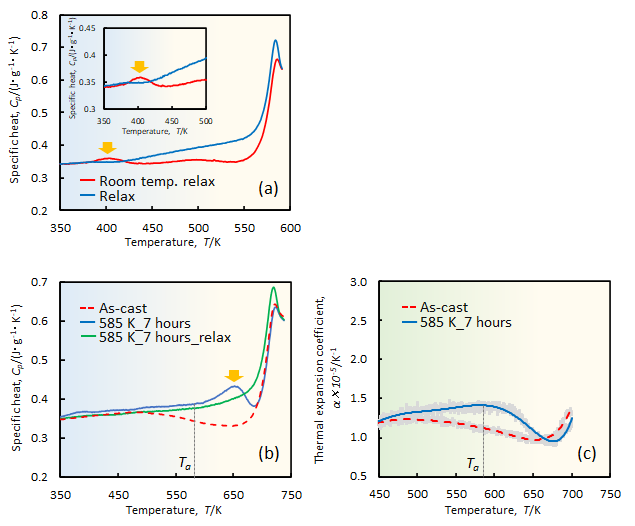


Supplementary Figure S1: *C_p_* data of two as-cast samples that show different thermal histories initially. *C_p_* curves of each relaxed state are also exhibited.

Supplementary Figure S2: *C_p_* curves of HPHTed_Relax samples, whose annealing temperatures are both 730 K, but annealing times differ (2 and 5 min). Curve of second heating result of as-cast sample is also presented.

Supplementary Figure S3: (a) *C_p_* curves of room-temperature relaxed Pd_42.5_Cu_30_Ni_7.5_P_20_ metallic glass (*i.e.*, prepared 3-mm glassy rod sample a few months before *C_p_* measurement) and its relaxed (annealed room-temperature relaxed state at 593 K for 2 min followed by 20 K/min cooling) data. Upper inset is a magnification image around the endothermic signal. (b) *C_p_* curves of annealed (*T_a_* = 585 K for 7 h) and relaxed (annealed at 740 K for 2 min followed by 20 K/min cooling) states. The as-cast data are shown as a reference. (c) *α* curve of annealed state (*T_a_* = 585 K for 7 h) and as-cast data.
